# Insulinoma with peripheral neuropathy: a case report

**DOI:** 10.1186/s13256-023-03963-5

**Published:** 2023-06-13

**Authors:** Marco Aurélio Vinhosa Bastos, Iago da Silva Caires, Renata Boschi Portella, Rogério Nascimento Martins, Ronald Reverdito, Stephen Reverdito, Nilson Moro

**Affiliations:** 1 Faculdade de Medicina, Universidade Federal de Mato Grosso do Sul, Av. Senador Filinto Muller, s/n Cidade Universitária, Campo Grande, MS 79070-900 Brazil; 2 Hospital Universitário Maria Aparecida Pedrossian, Universidade Federal de Mato Grosso do Sul, Campo Grande, MS 79070-900 Brazil; 3 Serviço de Endoscopia Digestiva, Hospital Universitário Maria Aparecida Pedrossian, Universidade Federal de Mato Grosso do Sul, Campo Grande, MS 79070-900 Brazil; 4 Hospital Santa Casa de Campo Grande, Campo Grande, MS 79002-251 Brazil; 5 Hospital São Julião, Campo Grande, MS 79017-200 Brazil

**Keywords:** Insulinoma, Hypoglycemia, Peripheral neuropathy, Lower limbs

## Abstract

**Background:**

Insulinomas are rare neuroendocrine tumors that typically present with hypoglycemic crises. Peripheral neuropathy is an uncommon complication of insulinoma. Most clinicians expect peripheral neuropathy symptoms to reverse completely after the insulin-secreting tumor is resected, but this may be a misassumption.

**Case report:**

We report a case of a 16-year-old Brazilian boy with clonic muscle spasms of the lower limbs for almost one year. Disabling paraparesis and confusional episodes had progressively set in as well. There were no sensorial abnormalities in the lower limbs, upper limbs or cranial nerves. An electromyography revealed a motor neuropathy of the lower limbs. The diagnosis of insulinoma was established as serum insulin and C-peptide concentrations were inappropriately normal during spontaneous episodes of hypoglycemia. Following a normal abdominal magnetic resonance scan, the imaging work-up continued with an endoscopic ultrasound, which localized the tumor at the pancreatic body-tail transition. Once localized, a prompt surgical removal (enucleation) of the tumor was undertaken, leading to an immediate and complete resolution of hypoglycemia. The time length between the onset of symptoms and tumor resection was 15 months. After surgery, the symptoms of peripheral neuropathy of the lower limbs showed a slow and only partial improvement. At a two-year follow-up after surgery, although being able to lead a normal and productive life, the patient still reported symptoms of reduced muscular strength in the lower limbs and a new electroneuromyography analysis showed chronic denervation and reinnervation in the legs’ muscles—indicating chronic neuropathic injury.

**Conclusion:**

The events of this case reinforce the importance of an agile diagnostic work-up and spry definitive treatment for patients with this uncommon disease, enabling the cure of neuroglycopenia before permanent bothersome complications ensue.

## Introduction

Hypoglycemia is an uncommon clinical situation except in individuals who use drugs that lower plasma glucose levels, particularly insulin or insulin secretagogues, to treat diabetes mellitus. Symptoms of hypoglycemia, which prompt the behavioral defense of food ingestion, typically develop at a mean plasma glucose concentration of approximately 55 mg/dL. Glucose is an obligate metabolic supply for the brain under physiological conditions. Because the brain cannot synthesize glucose, use alternative fuels effectively, or stock more than a few minutes of reserve as glycogen, maintenance of brain function requires nearly continuous supply of glucose from the circulation. Redundant glucose counterregulatory mechanisms normally effectively impede or rapidly correct hypoglycemia [1].

Symptoms of hypoglycemia can be classified as neuroglycopenic (the result of brain glucose deprivation per se) and autonomic (mainly resulting from the physiological changes caused by the sympathoadrenal discharge elicited by hypoglycemia). In fact, some autonomic symptoms due to hypoglycemia are adrenergic (such as palpitations, tremor, and arousal/anxiety), whereas others are cholinergic (such as sweating, hunger, and paresthesia). Neuroglycopenic symptoms range from behavioral changes, fatigue, and confusion to seizure and loss of consciousness. Awareness of hypoglycemia is mainly the result of the perception of autonomic symptoms by oneself, but neuroglycopenic manifestations are often observable by others [1].

There are different degrees of hypoglycemia. Patients with mild hypoglycemia experience symptoms such as hunger, sweating, headache, lack of concentration, irritation, tremor, muscle weakness and visual disturbances; they can elevate the blood glucose level through self-administration of glucose. Patients with moderate hypoglycemia have more severe symptoms and need help to treat the occurrence. Patients affected by severe hypoglycemia lose consciousness and may have convulsions. They depend upon assistance such as glucose injections to regain consciousness [1]. Without help, patients in this condition may die because profound and prolonged hypoglycemia can cause brain death [2].

Investigation of hypoglycemia is recommended in patients in whom Whipple’s triad is observed. This triad consists in symptoms and/or signs indicative of hypoglycemia, a low plasma glucose concentration, and resolution of those symptoms/signs after the plasma glucose concentration is raised. When the cause of hypoglycemia is not evident (in other words, when there are no clues to specific disorders—drugs, critical illnesses, hormone deficiencies, and so on), the following plasma analytes should be measured during an episode of spontaneous hypoglycemia: glucose, insulin, C-peptide, proinsulin, hypoglycemic agents, and insulin antibodies. A 48-hour supervised fast is often utilized to facilitate the occurrence of spontaneous hypoglycemia [1].

In normal physiological conditions, at a mean plasma glucose concentration ≤ 55 mg/dL, insulin secretion is suppressed virtually completely, plasma insulin levels are < 3 µU/mL, C-peptide levels are < 0.6 ng/mL, and proinsulin levels are < 5.0 pmol/L. The key pathophysiological feature of endogenous hyperinsulinism is the failure of insulin secretion to fall to very low levels as plasma glucose concentrations fall to hypoglycemic rates. Plasma insulin, C-peptide, and proinsulin concentrations need not be high relative euglycemic values, but only inappropriately high in the setting of low fasting plasma glucose concentrations [1].

In a patient with documented endogenous hyperinsulinemic hypoglycemia, negative screening for oral hypoglycemic agents, and no circulating insulin antibodies, the hypothesis of an insulinoma should be considered [1]. Insulinomas are the most common cause of endogenous hyperinsulinemic hypoglycemia in nondiabetic adult individuals, with an incidence of 1–3 per million per year. Insulinomas are mostly benign (more than 90%) and intrapancreatic. Approximately 85% are solitary, 6–13% are multiple. Familial types (5–10% of the cases) occur predominantly in association with type 1 multiple neuroendocrine syndrome (MEN-1). The clinical picture is characterized by symptoms of hypoglycemia secondary to hyperinsulinemia, typically in the morning (after an overnight fast) and triggered by physical exercise. Once a biochemical diagnosis of an insulinoma is established, localization procedures are performed. Due to their small size (82% < 2 cm, 47% < 1 cm), insulinomas may be difficult to localize. Computed tomography (CT) and magnetic resonance imaging (MRI) are widely available and are noninvasive localizing methods, but endoscopic ultrasound has greater accuracy for localizing tumors. Definitive treatment consists of surgical resection of the tumor. The recurrence rate after surgical resection is 7% for patients without MEN-1 and 21% for those with MEN-1. Long-term survival is the rule for individuals who have undergone successful insulinoma removal [3–6].

In the management of hypoglycemic episodes, seemingly complete neurological recovery after the glucose level is raised is the rule. However, neuropathological studies on material from patients experiencing prolonged and severe hypoglycemia demonstrate that neurons in both the central and peripheral nervous systems can be permanently affected in such cases [2].

We report the case of a male teenager with insulinoma and motor neuropathy of the lower limbs. We discuss the incidence and pathophysiology of this rare disease with an unusual complication. This case report was written in accordance to CARE guidelines [7]. The patient and his legal guardian gave an informed consent and the study was approved by the Ethics Committee of the Universidade Federal de Mato Grosso do Sul—UFMS (Aug 10, 2021—CAAE number 50027421.0.0000.0021).

## Case presentation

A 16-year-old Brazilian boy was referred to an endocrinologist physician in Vital Policlínica, a private multi-specialty ambulatory clinic in Campo Grande, Midwest Brazil, in September 2019, for assessment of cognitive dysfunction and lower limbs weakness. The teenager, who lived in a neighboring city and was accompanied by his father, said that his symptoms began 1 year before, when he started experiencing frequent episodes of muscle spasms in his thighs and legs. His symptoms usually appeared after physical exercise and improved by drinking sweetened beverages. Few weeks after the onset of symptoms, he had consulted a local primary care physician who ordered some blood exams that documented hypoglycemia (Table 1 shows the relevant blood analysis results). According to his report, he was then advised solely to “eat better.” The patient had no past medical or surgical history. He denied smoking, alcohol consumption or any other harmful habits. He had a family history of essential hypertension and type 2 diabetes mellitus in his grandparents, although his parents were healthy.

**Table 1:** Blood analysis results of the patient

	Sep 2018	Jul 2019	Oct 2019	Nov 2019	Oct 2021	Reference interval
Hemoglobin (g/dL)			14.1		14.9	(13.5–17.5)
White blood cell count (/mm^3^)			4700		4420	(4000–11,000)
Platelet count (/mm^3^)			153,000		176,000	(140,000–400,000)
ESR (mm)			28			(10–30)
Glucose (mg/dL)	46.0	85.0	38.1		115.0	(70–99)
Insulin (µU/mL)	12.5		13.5		5.8	(1.9–23.0)
C-peptide (ng/mL)	1.65		1.9		1.4	(1.1–4.4)
Hemoglobin A1C (%)	4.5		4.6		5.6	(< 5.7)
Creatinine (mg/dL)	0.71	1.0	0.94		0.77	(0.5–1.2)
Urea (mg/dL)	28.0	43.0				(10–45)
Uric acid (mg/dL)			3.7		6.4	(2.5–7.0)
Sodium (mmol/L)			144.0		131.0	(132–148)
Potassium (mmol/L)			4.6		4.6	(3.5–5.5)
Calcium (mg/dL)			8.9		9.6	(8.0–10.5)
Magnesium (mg/dL)			1.8		2.2	(1.6–2.6)
ALT (U/L)	21.0	68.0	34.0		15.0	(11–45)
AST (U/L)	24.0	71.0	36.2		20.0	(11–39)
Gamma‐glutamyl transferase (U/L)		15.0	30.8		10.0	(7.0–58.0)
Total bilirrubin (mg/dL)			1.43		1.76	(< 1.0)
Direct bilirrubin (mg/dL)			0.33		0.58	(< 0.4)
Indirect bilirrubin (mg/dL)			1.1		1.18	(< 0.6)
TSH (mcIU/mL)			1.81		0.90	(0.38–5.33)
Free T4 (ng/dL)			0.9			(0.89–1.76)
Cortisol (mcg/dL)			14.9		10.1	(6.7–22.6)
ACTH (pg/mL)			67.8			(< 46.0)
Prolactin (ng/mL)			13.3			(2.7–17.7)
IGF-1 (ng/mL)			477.0		256.0	(137–428)
Total testosterone (ng/dL)			328.2		391.7	(175–781)
Noradrenaline (pg/mL)				18.5		(< 460.0)
Adrenaline (pg/mL)				< 15.0		(< 90.0)
Dopamine (pg/mL)				< 15.0		(< 30.0)
Antinuclear antibody		Negative				(Negative)
Anti-Sm antibody		Negative				(Negative)
Anti-Ro/SS-A antibody		Negative				(Negative)
Anti-La/SS-B antibody		Negative				(Negative)
Rheumatoid factor		Negative				(Negative)
Anti-HIV		Negative				(Negative)
Anti-HCV					Negative	(Negative)
HBsAg					Negative	(Negative)
Anti-HAV IgM					Negative	(Negative)
Vitamin B12 (pg/mL)					184.0	(130.0–868.0)
25-hydroxyvitamin D (ng/mL)		35.1	44.9			(> 20.0)
Total cholesterol (mg/dL)			151.0		105.0	(< 200.0)
High density lipoprotein (mg/dL)			55.0		43.1	(> 40.0)
Low density lipoprotein (mg/dL)			74.8		47.0	(< 100.0)
Triglycerides (mg/dL)			106.0		78.0	(< 150.0)

*ESR* Erythrocyte sedimentation rate, *ALT* Alanine transferase, *AST* Aspartate transferase, *TSH* Thyroid-stimulating hormone, *Free T4* Free thyroxine, *ACTH* Adrenocorticotropic hormone, *IGF-1* Insulin-like growth factor 1, *Anti-HIV* Human immunodeficiency virus antibody test, *Anti-HCV* Hepatitis C virus antibody test, *HBsAg* Hepatitis B surface antigen, *Anti-HAV* Hepatitis A virus antibody test

In the following months, muscle spasms of the lower limbs recurred frequently. Additionally, lower limbs weakness progressively worsened. He kept on drinking sweetened beverages to control symptoms. Around one year after symptoms’ onset, the clinical picture deteriorated as he started having episodes of confusion, unusual behavior, and aggressiveness, mostly in the middle of the night. In these occasions, his father needed to take him hastily to the hospital to receive glucose intravenous infusions, which brought him back to a normal state. Back then, the worsening of symptoms made him abandon his initial nursing studies at the University and, during the night, he had to wake every two hours by an alarm clock and eat something to avoid hypoglycemic crises.

His parents decided to seek help from a neurologist physician. The first neurologist that attended him conferred him the clinical diagnosis of epilepsy without undertaking any complementary exam. The patient then started taking the anticonvulsants carbamazepine and phenytoin, which led to slight elevation in liver enzymes but no relief from the confusional episodes. Hence, they decided to seek a second opinion from another neurologist physician. The second neurologist who attended him undertook a neurological physical examination, which demonstrated lower limbs paresis with proximal strength grade 3, grade 3− for feet dorsiflexion and grade 3+ for plantar flexion, abolished patellar and Aquilian reflexes, with difficulty on standing upright and walking, without cranial nerve, sensitive or upper limbs abnormalities. Specifically, the examination of the sensory points within each side of the body did not demonstrate a sensory level and no sphincter abnormalities were noted, speaking against spinal cord injuries. Also, he had a normal brain computed tomography scan. Nerve conduction tests were performed on the lower limbs only, revealing mild reduction in amplitude of compound muscle action potentials and mild reduction in motor conduction velocities (CV). Sensory latencies and amplitude of the sensory action potentials were normal (Table 2). Needle electromyography revealed signs of chronic neurogenic changes in the motor unit potentials of gastrocnemius, anterior tibialis, posterior tibialis, and vastus lateralis muscles, bilaterally. These findings accorded with a recently developed axonal peripheral polyneuropathy of the lower limbs, with a motor predominance.

**Table 2:** Nerve conduction studies of the patient

Nerve (muscle studied)	Variable	Initial study		Follow-up study		Normal value
		Right	Left	Right	Left	
Motor nerves						
Median (abductor pollicis brevis—wrist)	Distal latency (ms)	Not done		3.3	3.4	≤ 4.5
	Amplitude (mV)			9.9	13.5	≥ 5.9
	Conduction velocity (m/s)			57.9	58.6	≥ 50
Ulnar (abductor digiti minimi—elbow)	Distal latency (ms)	Not done		7.3	6.9	≤ 3.7
	Amplitude (mV)			5.1	5.7	≥ 7.9
	Conduction velocity (m/s)			61.4	61.9	≥ 50
Fibular (extensor digitorum brevis—ankle)	Distal Latency (ms)	3.4	4.1	3.2	4.6	≤ 6.5
	Amplitude (mV)	3.1	3.0	4.6	5.4	≥ 4
	Conduction velocity (m/s)	42.2	40.5	43.9	43.0	≥ 42
Tibial (abductor hallucis—medial malleolous)	Distal latency (ms)	4.6	4.4	3.8	4.8	≤ 5.1
	Amplitude (mV)	5.0	6.7	7.2	8.6	≥ 5.8
	Conduction velocity (m/s)	41.9	43.4	41.5	47.9	≥ 38
Sensory nerves						
Median (second digit, wrist)	Onset latency (ms)	Not done		2.4	2.3	≤ 3.3
	Peak latency (ms)			3.2	3.3	≤ 4
	Amplitude (µV)			15.1	18.7	≥ 11
Ulnar (fifth digit)	Onset latency (ms)	Not done		2.2	2.0	≤ 3.1
	Peak latency (ms)			2.9	2.8	≤ 4
	Amplitude (µV)			12.2	11.3	≥ 10
Medial antebrachial cutaneous	Onset latency (ms)	Not done		1.6	1.6	≤ 2.2
	Peak latency (ms)			2.3	2.3	≤ 2.6
	Amplitude (µV)			15.8	22.3	≥ 4
Sural	Onset latency (ms)	1.7	2.0	2.3	2.3	≤ 3.6
	Peak latency (ms)	2.8	3.2	3.1	3.4	≤ 4.5
	Amplitude (µV)	8.2	7.6	16.0	13.1	≥ 5

Abnormal values are underlined

**Fig. 1:**
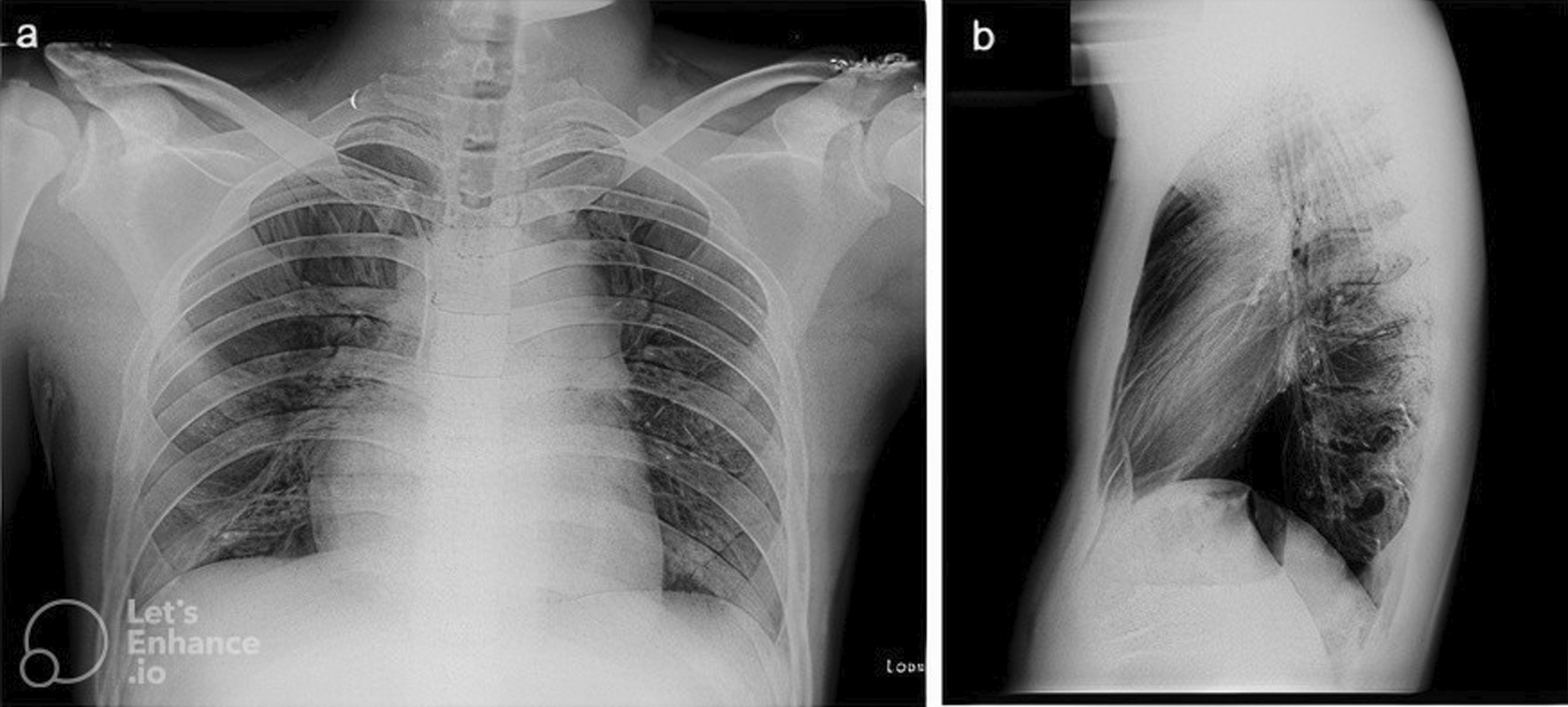
Patient’s normal chest X-ray. **A** Posteroanterior (PA) view. **B** Lateral view

The neurologist referred him to an endocrinologist physician who diagnosed insulinoma on the basis of inappropriately normal serum insulin and C-peptide concentrations during a spontaneous episode of hypoglycemia (Table 1). At the initial consultation with the endocrinologist, the patient had a normal mental status, his capillary blood glucose was 71 mg/dL and his physical examination was as follows: radial pulse 88 beats/min, arterial blood pressure 120/80 mmHg, axillary temperature 36.5 °C, weight 90 kg, height 195 cm, and body mass index 23.7 kg/m^2^. Muscle strength of lower limbs was not assessed at that occasion, but muscle mass of the lower legs, by inspection, appeared slightly decreased. The rest of the clinical examination was unremarkable. His chest X-ray was normal as well (Fig. 1).

When managing a case of a pancreatic neuroendocrine tumor, the clinician should rule out the possibility of multiple endocrine neoplasia type 1 (MEN-1), which is an hereditary condition predisposing the development of multiple endocrine cell tumors [3–6]. The most common tumors seen in MEN-1 involve the parathyroid gland, pancreatic islet cells, and pituitary gland. Epidemiological data indicate that when the patient affected with MEN-1 has a pituitary tumor, this tumor secretes prolactin in about 90% of cases [8]. In our patient, the hypothesis of MEN-1 was ruled out as there was no family history of endocrine neoplasia, prolactin levels were normal, calcemia was normal (ruling out hyperparathyroidism), and there was no complaint of epigastralgia or history of peptic ulcer (speaking against the presence of a concomitant gastrinoma).

The patient’s blood test results, except for glucose, insulin, and C-peptide, were predominantly normal. Nevertheless, there were some minor abnormalities that require some comments. It is noteworthy that the patient had mild hyperbilirubinemia, mainly unconjugated bilirubin (Table 1). This finding was present both before and after the insulinoma resection. On the other hand, plasma aminotransferases concentrations were normal, except during a short period before surgery when the patient was taking anticonvulsant medications. We believe that our patient has Gilbert’s Syndrome, an autosomal recessive hereditary condition with a prevalence rate of 4%–16%, that causes reduced glucuronidation of bilirubin within the liver. This abnormality results in unconjugated hyperbilirubinemia (bilirubin is typically below 4 mg/dL) and may lead to recurrent episodes of jaundice. The condition is more frequent in males and usually is diagnosed during the adolescence. Some triggers have been identified that precipitate unconjugated hyperbilirubinemia and jaundice in patients with Gilbert’s syndrome, prolonged fasting being one of these triggers. Other predisposing factors include: dehydration, febrile illnesses, menstruation, and strenuous exercise [9, 10]. It is remarkable that the patient in the present report did not present jaundice; moreover, no significant change in bilirubin were noted when comparing its levels before and after the insulinoma resection. Individuals with Gilbert’s syndrome have an increased incidence of gallstones, but it is well known that carriers are not at significant risk for progressive liver disease; the condition does not require treatment and has an excellent prognosis [9–11]. Interestingly, it has been discovered that bilirubin has antioxidant action and mild unconjugated hyperbilirubinemia may have beneficial effects, decreasing the incidence of atherosclerosis and of several types of cancer [11].

Other blood analytes with altered levels were adrenocorticotropic hormone (ACTH) and insulin-like growth factor-1 (IGF-1) in a sample collected during an episode of severe hypoglycemia (Table 1). We have interpreted these slightly increased levels of ACTH and IGF-1 as a normal defense mechanism, provided that they are counterregulatory hormones and the patient had severe hypoglycemia when the blood sample was collected. A new blood sample collected after insulinoma resection showed normal cortisol and IGF-1 concentrations.

**Fig. 2:**
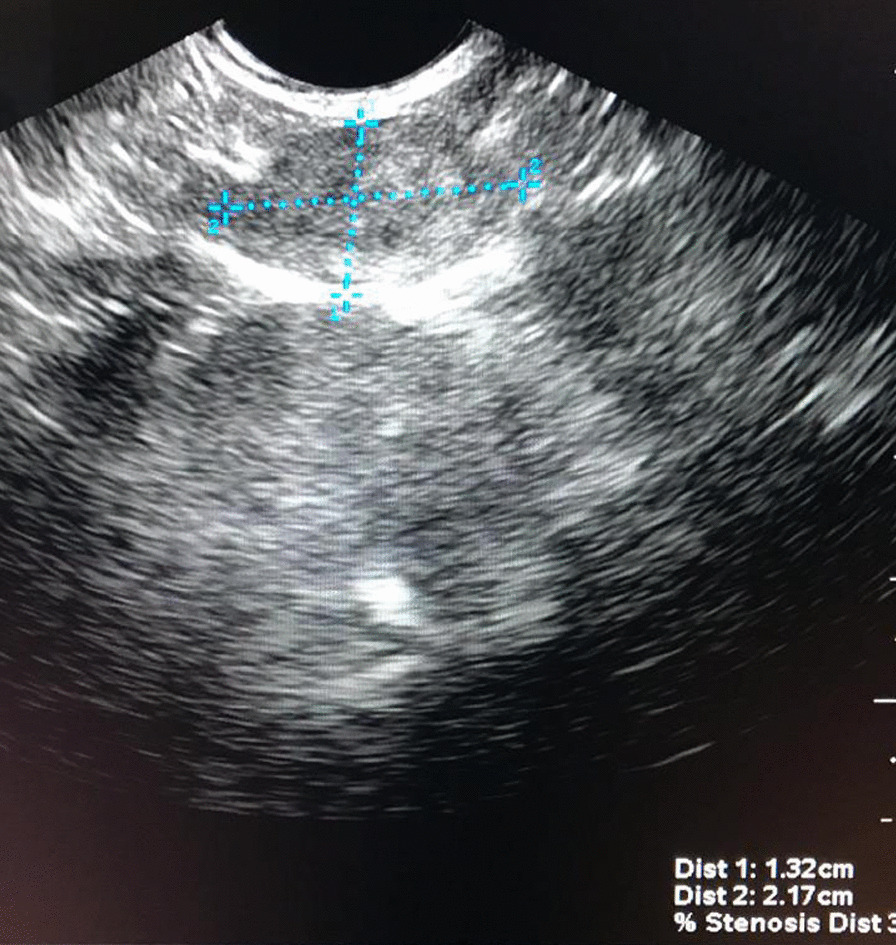
Endoscopic ultrasound of the patient showing a pancreatic nodule

While the radiological investigation was underway, the patient started taking verapamil 80 mg bid, but this was not effective to prevent hypoglycemic crises either. The patient underwent an abdominal MRI study (1.5 T MR scanner—Phillips MR Systems Achieva) with intravenous contrast using T1-weighted and T2-weighted images with and without fat-suppression, T1-weighted gradient-echo in-phase and out-of-phase, and short-tau inversion-recovery (STIR) sequences, with 3.0-mm slice thickness. The radiologic report of the abdominal MRI was of a normal exam, so the tumor localization plan advanced to an endoscopic ultrasound, which revealed a 2.2 × 1.3 cm oval homogeneous peripancreatic nodule, in the body-tail transition (Fig. 2). The core biopsy demonstrated a neuroendocrine pancreatic tumor.

**Fig. 3:**
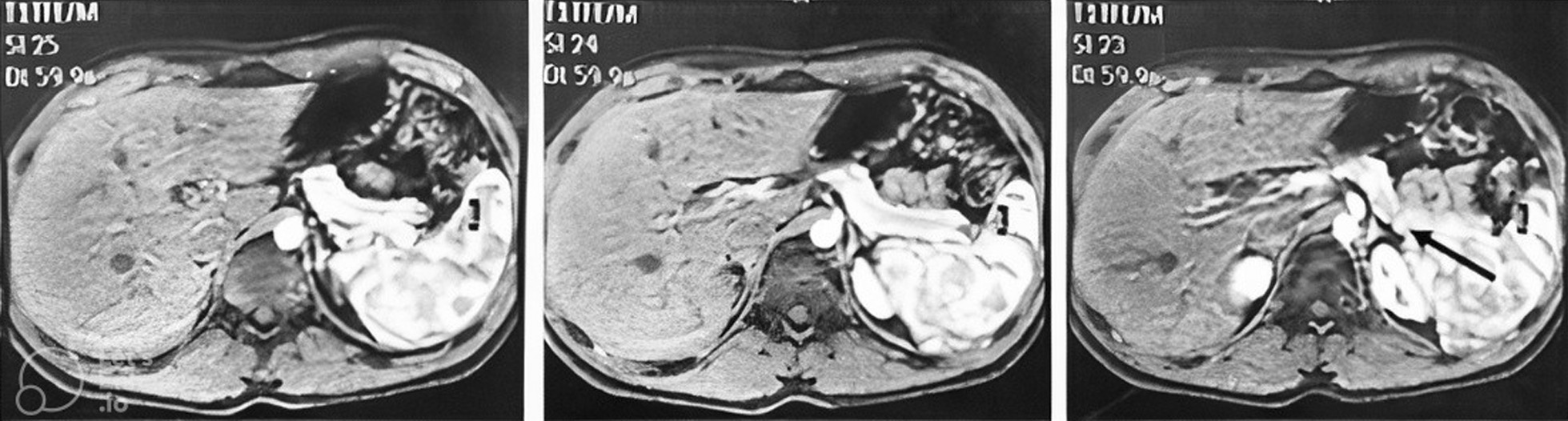
Magnetic resonance imaging of the patient’s pancreas. Arrow indicates the probable nodule in the body–tail transition

For the preparation of the present manuscript, we retrospectively assessed the patient’s MRI. Guided by the endoscopic ultrasound findings, we noticed that the probable image of the nodule is visible at the corresponding location (Fig. 3).

**Fig. 4:**
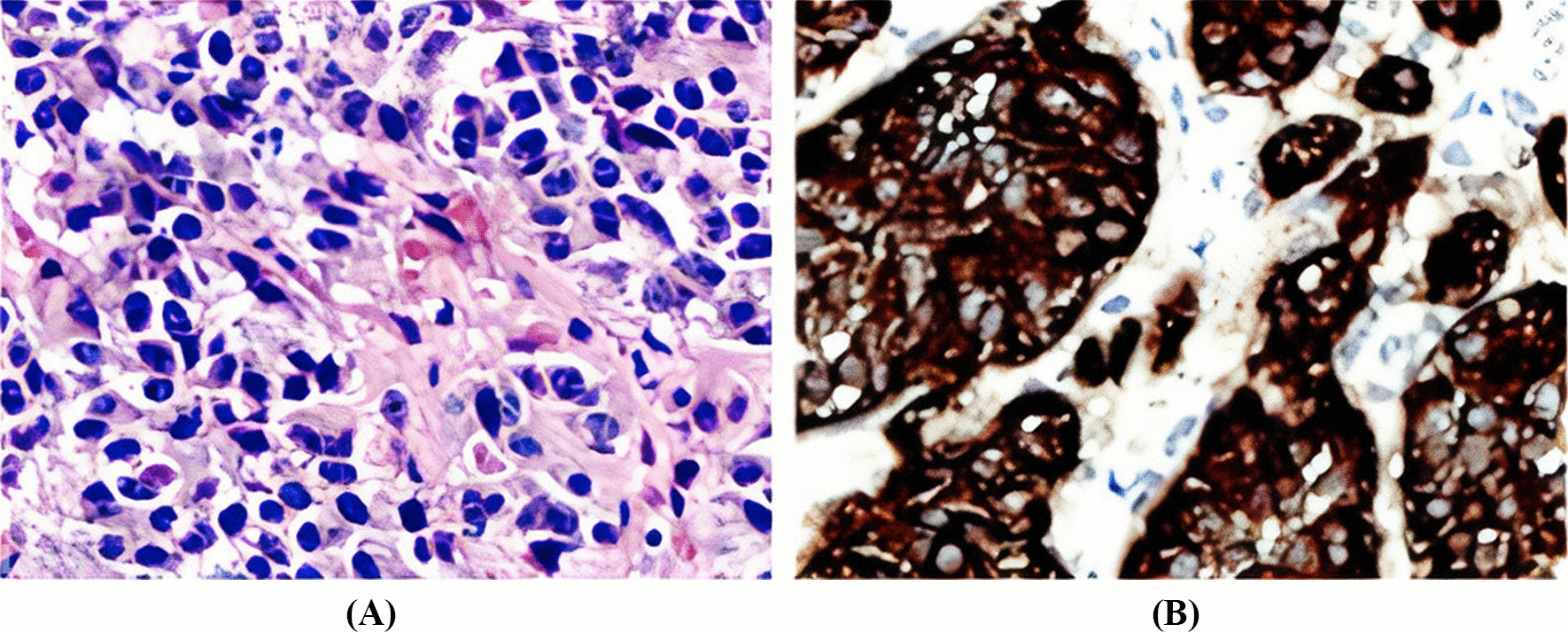
Anatomopathological analysis of the resected pancreatic nodule. **A** Hematoxylin and eosin stained sections showing a neuroendocrine tumor, grade 1. **B** Immunohistochemistry study showing tumor cells stain for synaptophysin

The patient was referred to a hepatobiliary surgeon and submitted to a laparotomy, with pancreatic nodulectomy and resection of abdominal lymph nodes (two intercavoaortic and four splenic lymph nodes). The anatomopathological analysis showed a 1.5 cm well differentiated neuroendocrine pancreatic tumor (grade 1), without vascular or perineural invasion, mitotic count 02/10 CGA, and lymph nodes without malignancy. The immunohistochemistry panel demonstrated histological aspects of a well differentiated neuroendocrine neoplasm, represented by intermediate and monomorphic cells, in a solid, organoid, and extensively hyalinized arrangement in the pancreatic parenchyma. Neuroendocrine differentiation was established by the coexpression of chromogranin A and synaptophysin, positively immunoexpressing insulin. The findings correspond to neuroendocrine tumor (NET) grade 2 [World Health Organization (WHO)—intermediate grade]. The markers for biliopancreatic differentiation and solid pseudopapillary neoplasia were non-reactive (Fig. 4).

From the surgery onward, the patient had no more hypoglycemic crises nor muscle spasms in the lower limbs. All the medications were withheld. For around 4 months he regularly practiced weightlifting exercises at the gym, which he considered helpful. Six months after surgery, the patient had improved deambulation, with slight distal lower limb weakness, mostly at the left side. The lower limbs examination demonstrated proximal muscular strength grade 4+ , grade 4− for the left foot dorsiflexion, grade 4 for the right foot dorsiflexion and grade 4+ for bilateral plantar flexion, with patellar reflexes hypoactive and abolished Aquilian reflexes.

Later, at a 2-year follow-up visit, he was leading a normal and productive life (including cycling 8 km every day, working in construction, and studying for a nursing degree), but still reported reduced muscular strength in the lower limbs. His weight was 76 kg (body mass index 19.7 kg/m^2^). New nerve conduction tests were performed, this time both on the upper and the lower limbs. The amplitude of compound muscle action potentials and motor CV were both normal, indicating a partial improvement in comparison with the tests performed two years before. However, the needle electromyography was unchanged in comparison with the test performed two years before, with the same lower limb muscles showing signs of chronic neuropathy.

As the patient had no complaints related to the upper limbs and the physical exam of the upper limbs was normal, we anticipated that the nerve conduction tests of the upper limbs would be normal. Contrary to our expectations, the tests revealed reduction in amplitude of compound muscle action potentials and mild increase in distal motor latencies in the ulnar nerves. In line with this, mild chronic neurogenic changes were present in muscles innervated by ulnar and median nerves, as revealed by the needle electromyography.

**Fig. 5:**
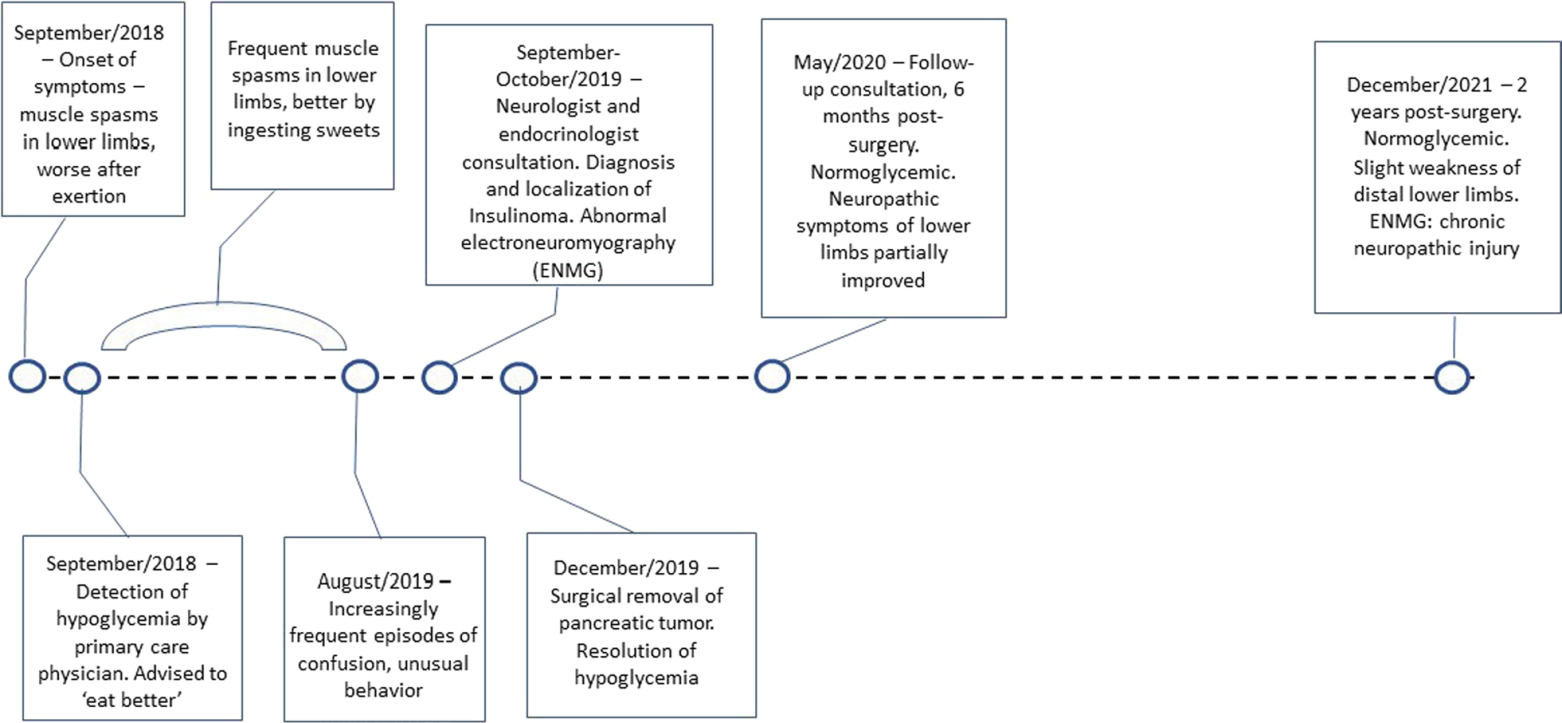
Timeline of the main events in the medical history of the patient

Noteworthy, the investigation and treatment of the patient was challenging because he had no private health insurance, and because of the complex complementary exams and surgical procedures that were necessary. Nevertheless, the patient’s family and most of the health professionals were remarkably committed to offer him a proper management. Despite these efforts, from the onset of symptoms until the surgery it took longer than a year (Fig. 5).

In his most recent medical consultation, when asked to talk about his illness experience, the patient expressed himself in the following terms: “Back then (when I had the tumor), I didn’t have a life. I could not sleep, I had to stay always alert. I didn’t have a glucometer, so I knew that it was very risky and that any moment I might need to go to hospital to receive a glucose injection. Since I was submitted to surgery, things are not 100% but they are very well. I restarted my evening nursing studies at the University, I intend to be an Emergency Room Nurse! I am employed as a construction worker for a year now, so that I can pay for my studies.”

## Discussion

We have reported the case of an individual with peripheral neuropathy associated with a pancreatic insulinoma. First, it is worth mentioning that preoperative localization studies for insulinomas include both noninvasive and invasive imaging techniques. The noninvasive procedures available include transabdominal ultrasonography, spiral computed tomography, magnetic resonance imaging, scintigraphy, and positron emission tomography. Invasive examinations chiefly include endoscopic ultrasonography or a selective arterial calcium stimulation test with hepatic venous sampling [3, 5]. In the present case, the MRI of the abdomen did not localize the insulinoma despite the tumor being > 1 cm in diameter. The explanation for this may be the peripancreatic and perisplenic location of the tumor, close to the tail of the pancreas. Accessory spleens are frequently found in this anatomical region (with a prevalence of up to 30% in the general population) which can act as confounding factors for radiologists [12, 13]. Hence, this is of utmost importance to continue the investigation with other imaging methods (e.g., endoscopic ultrasound) when the index of clinical suspicion for insulinoma is high. The sensitivity of endoscopic ultrasound for localization of insulinoma is 75% [14]. Endoscopic ultrasonography was very useful in the work-up of our patient, promptly localizing the pancreatic nodule.

As mentioned before, neurons in both the central and peripheral nervous systems can be affected by prolonged and severe hypoglycemia. With regard to the effects on the central nervous system, pathological studies on material from patients with hypoglycemia (caused either by exogenous insulin, hypoglycemic medication or insulinoma) demonstrate that neurons in the neocortex, hippocampus, thalamus, and hypothalamus are affected most. Neurons in the brain stem, the cerebellum and the spinal cord are more resistant. The histopathological picture includes axonal degeneration and demyelination, but it is not clear whether the demyelination was primary or secondary to axon loss [2].

The cellular mechanisms behind hypoglycemic neuropathy involve ATP depletion, loss of ionic homeostasis, mitochondrial damage, and excitotoxicity. Excitotoxicity is the pathologic process in which exacerbated or prolonged activation of excitatory neurotransmitters—primarily glutamate—receptors start a cascade of neurotoxicity that ultimately leads to neuronal death [2].

Peripheral neuropathy due to hypoglycemia typically manifests as a distal axonopathy preferentially affecting large motor axons. In line with this concept, there are reports of insulinoma patients who develop a distal symmetric predominantly motor neuropathy, exhibiting axonal degeneration in peripheral nerves. The typical clinical presentation begins with persistent dysesthesia, progressing to distal amyotrophy and reduction of deep distal tendon reflexes. Reports of neurophysiological analyses have revealed slowed muscle nerve conduction velocities, and there is electromyographic evidence for denervation of skeletal muscle. It has been noted that primary sensory neurons have greater tolerance to hypoglycemia. Motor nerve fibers are thicker than sensory fibers and there is evidence that the former regenerate less than the latter [2]. In addition, as the sensations conveyed by the sensory fibers are of different types, this may contribute to the deficits being less perceived by the patients, compared with the motor deficits.

The reports available in the literature on peripheral neuropathy due to insulinoma are sparse. Tintoré et al. (1994) reported 30 patients [15], Heckmann et al. (2000) reported 34 patients [16], and Striano et al. (2003) reported 40 patients [17]. Data indicate that an upper limbs predominance of symptoms is more common than a lower limbs predominance [2]. For instance, of the 34 patients mentioned by Heckmann et al., 11 had entirely motor neuropathy, 3 predominantly or completely sensory, and the remaining 20 had a sensorimotor pattern [16]. With respect to localization, 9 of the cases were described as upper limb predominant, 2 as lower limb predominant and, in 23 cases, no predominance was established [16].

With respect to the cellular mechanisms involved in distal axonopathy-like picture seen in hypoglycemic neuropathy, this is clear that it may be difficult to maintain a long axon if energy supply is so deficient that protein synthesis is reduced. Additionally, the maintenance of the distal part of the axon may be compromised if the energy-consuming axonal transport is impaired. Finally, hypoglycemia may have detrimental effects on mitochondria (e.g., altered mitochondrial membrane permeability) at the level of axon, affecting energy production in the axon itself, in the associated Schwann cells, in the local blood vessels, and/or in the perineurial sheath [2].

In the investigation of our patient’s peripheral neuropathy, other causes of peripheral neuropathy were ruled out, mainly through blood tests. Renal, liver and thyroid function were normal, serology for human immunodeficiency (HIV) and for viral hepatitis were negative, antinuclear factor and rheumatoid factor were negative, and vitamin B12 levels were within the normal range. A lumbar puncture was not performed because his clinical picture did not suggest the presence of an autoimmune demyelinating neuropathy [18]. In such cases (for example, in chronic inflammatory demyelinating polyneuropathies) there is a characteristic course of progressive weakness and impaired sensory function in the legs and arms [18]. Our patient’s clinical pattern was one of motor deficits ensuing after each episode of severe hypoglycemia. Then, after the insulinoma was completely resected, there was a small improvement, followed by stabilization of the patient’s motor function. The clinical facts indicate that hypoglycemia was the causal factor for the patient’s peripheral neuropathy, and apparently its intensity and duration were of an extent sufficient to cause neuronal death (motor neurons), given that even after the complete removal of the causal factor there was no resolution of motor dysfunction. The fact that the patient did not adhere to a more extensive physical therapy program may have contributed to the insufficient improvement of the peripheral neuropathy.

Gabapentin, pregabalin, and tricyclic antidepressants are the pharmacological agents with more evidence of benefit in the treatment of peripheral neuropathy when there are painful symptoms [19]. However, the patient in the present report did not have painful symptoms, so these drugs were not used. On the other hand, there is some evidence that vitamin B12 and B vitamin complex supplementation may improve sensory symptoms due to peripheral neuropathy [20, 21]. As previously mentioned, sensory symptoms were very scarce in our patient, notwithstanding vitamin B12 supplementation was tried for a few months due to the chronicity of his neuropathic symptoms. However, he reported no improvements in sensory or motor symptoms following this treatment.

## Conclusion

The case here described illustrates an atypical presentation of insulinoma, in other words, a peripheral neuropathy causing a predominantly motor impairment of the lower limbs. Most clinicians would expect peripheral neuropathy symptoms to reverse completely after the resection of an insulin-secreting tumor, but this may be a misassumption. In our patient, the peripheral neuropathy was only partially reversed after the surgical removal of the insulinoma, as shown in the clinical and electromyographic reassessment two years later. As we have reviewed, the extent to which the neurons are damaged varies depending on the duration and severity of hypoglycemia. The events of the presently reported case reinforce the necessity of an agile diagnostic work up and prompt surgical treatment for insulinoma patients, enabling the cure of neuroglycopenia before permanent bothersome complications ensue.

## Data Availability

Not applicable.
